# Probabilistic Assessment of High-Energy Acoustic Emission Events During Tensile Deformation of Thermally Exposed S235 Structural Steel

**DOI:** 10.3390/ma19214602

**Published:** 2026-10-30

**Authors:** Anna Adamczak-Bugno

**Affiliations:** Faculty of Civil Engineering and Architecture, Kielce University of Technology, al. Tysiąclecia Państwa Polskiego 7, 25-314 Kielce, Poland; aadamczak@tu.kielce.pl

**Keywords:** acoustic emission, S235 structural steel, thermal exposure, tensile deformation, high-energy events, exceedance probability, logistic regression, post-fire assessment

## Abstract

**Highlights:**

- Stage-specific RT thresholds identified upper-tail acoustic-emission energy events.
- Specimen-level probabilities accounted for the clustering of hits within specimens.
- Nominal furnace exposure at 700 and 1100 °C was associated with higher adjusted odds of high-energy events.
- Stage III showed the highest adjusted odds relative to the pre-yield stage.
- Channel differences confirmed the sensitivity of AE energy to the measurement path.

**Abstract:**

Thermal exposure can modify the tensile response of structural steel and the acoustic-emission (AE) activity recorded during deformation. This study presents a stage-resolved probabilistic analysis of high-energy AE events in S235 steel tested as received and after nominal furnace exposure to 700, 900, or 1100 °C for 40, 80, or 120 min, followed by still-air cooling. Twenty specimens with archived stage-resolved AE records were analysed from a 30-test mechanical programme. Each record was divided into pre-yield, yielding, plastic deformation to ultimate tensile strength, and post-UTS localization/fracture stages. High-energy events were defined using stage- and channel-specific thresholds derived from the 90th percentiles of room-temperature specimens. For Channel 1, the adjusted odds were higher after exposure to 700 and 1100 °C than at room temperature (OR = 5.36 and 6.71), whereas the 900 °C estimate was positive but uncertain (OR = 3.60; 95% CI: 0.93–13.93). Stage III showed higher odds than Stage I (OR = 3.54; 95% CI: 2.15–5.84). Analyses using 85th- and 95th-percentile thresholds preserved the positive temperature estimates and higher Stage II–III odds. Leave-one-specimen-out refits confirmed stable Stage II, Stage III, and 1100 °C associations, while 700 and 900 °C were more specimen-sensitive. The framework remains exploratory and measurement-configuration-specific; sparsely represented conditions are descriptive.

## 1. Introduction

Structural steel members exposed to elevated temperature may undergo changes in strength, ductility, yield behavior, and deformation capacity even when no severe geometric damage is visible after cooling [1,2,3,4,5,6,7]. The extent of these changes depends not only on the maximum temperature but also on the exposure duration, cooling conditions, initial material state, and subsequent loading path [1,2,3,4]. Consequently, the assessment of thermally exposed steel requires methods capable of identifying changes in the evolution of deformation rather than relying exclusively on final strength parameters.

The onset of ductile fracture in metallic materials can also be evaluated using stress-state-dependent fracture criteria calibrated against mechanical tests and implemented in finite element models. Recent applications to SUS304 stainless steel and QP980 sheet steel demonstrate the usefulness of such approaches for predicting fracture initiation under different loading and stress states [8,9]. These methods provide a complementary mechanical description of fracture but do not directly characterize the stochastic evolution of transient activity recorded during deformation.

Acoustic emission (AE) monitoring provides information about transient elastic waves generated during active deformation and damage processes. During tensile loading of low-carbon structural steel, AE activity may accompany dislocation motion, yielding, Lüders-band propagation, strain hardening, necking, and final fracture [10,11,12,13,14,15,16]. The recorded response is also affected by signal propagation, specimen geometry, sensor position, coupling, acquisition settings, and the applied detection threshold [17,18,19,20,21,22]. AE parameters therefore should not be interpreted as direct measurements of individual microstructural mechanisms. Nevertheless, when the testing configuration and acquisition settings are kept constant, changes in AE activity can provide comparative information about the deformation response of differently conditioned specimens.

Most AE investigations of metallic and composite materials use cumulative hit counts, cumulative energy, event rates, or descriptive statistics of individual signal parameters [11,13,23,24]. These measures are useful for identifying the overall level and temporal evolution of activity, but they do not directly describe the probability that an AE hit will exceed a physically or empirically defined energy level. This distinction is important because two specimens may generate similar numbers of hits while differing considerably in the proportion of relatively energetic signals. Conversely, high cumulative energy may be controlled by a small number of extreme observations and may therefore obscure the distribution of activity among specimens.

A probabilistic formulation can complement conventional AE descriptors by expressing energetic activity as an exceedance event. However, hit-level AE observations are not statistically independent because many hits originate from the same specimen and deformation stage. Treating every recorded hit as an independent experimental replicate would result in pseudoreplication and could substantially underestimate uncertainty [25,26,27]. A suitable analysis should therefore retain the specimen as the experimental unit or account explicitly for the clustering of hits within specimens [26,27]. It should also distinguish between deformation stages because the baseline AE energy distribution changes markedly between pre-yield loading, yielding, strain hardening, and post-UTS localization.

The present study applies such a stage-resolved probabilistic framework to AE data obtained during tensile testing of S235 structural steel subjected to different thermal-exposure conditions. The investigated states comprised room-temperature material and specimens exposed to 700, 900, or 1100 °C for 40, 80, or 120 min, followed by still-air cooling. The tensile response was divided using mechanically identified transition times into four stages: Stage I, preceding the lower yield point; Stage II, corresponding to yielding and the yield plateau; Stage III, covering plastic deformation from the end of the yield plateau to the ultimate tensile strength; and Stage IV, extending from UTS to fracture.

For each stage and measurement channel, a room-temperature reference threshold was derived from specimen-specific 90th percentiles of the logarithmically transformed absolute energy. The proportion of hits exceeding this threshold was subsequently determined for every available specimen. The analysis combined empirical cumulative distribution functions, specimen-level exceedance probabilities, bootstrap uncertainty intervals, condition-specific odds ratios, and a binomial logistic model with specimen-clustered robust standard errors [25,28]. Channel 1 was used for the primary analysis, whereas Channel 2 provided a complementary assessment of measurement-path sensitivity.

The objective was to determine whether thermal exposure was associated with changes in the probability of recording high-energy AE events during successive stages of tensile deformation. It was hypothesized that these changes would depend on both thermal condition and deformation stage and would be most pronounced during plastic deformation to UTS. The contribution of the study is therefore not another comparison of cumulative AE activity, but a specimen-aware probabilistic assessment of stage-dependent energy exceedance. Because the available numbers of specimens were unequal among exposure conditions, the analysis was designed as an exploratory assessment, with the strongest interpretation reserved for the better-represented conditions and for effects supported by the clustered model.

## 2. Materials and Methods

### 2.1. Material, Specimen Preparation, and Thermal Exposure

The investigation was conducted using S235 low-carbon structural steel supplied as a hot-rolled plate. The material designation was established from the available product documentation. Because detailed chemical-composition data were unavailable, the results are regarded as specific to the investigated steel product and should not be generalized to every steel heat classified as S235.

Flat tensile specimens were cut parallel to the rolling direction. The specimens had a symmetrically tapered reduced section, with the width decreasing from approximately 10 mm near the transition regions to 6 mm at the centre of the gauge section. The nominal thickness was approximately 5 mm, and the extensometer gauge length was 25 mm. The dimensions of each specimen were measured individually with a resolution of 0.01 mm. The minimum cross-sectional area measured after thermal exposure, cooling, and oxide-scale removal was used to calculate engineering stress.

**Figure 1:**
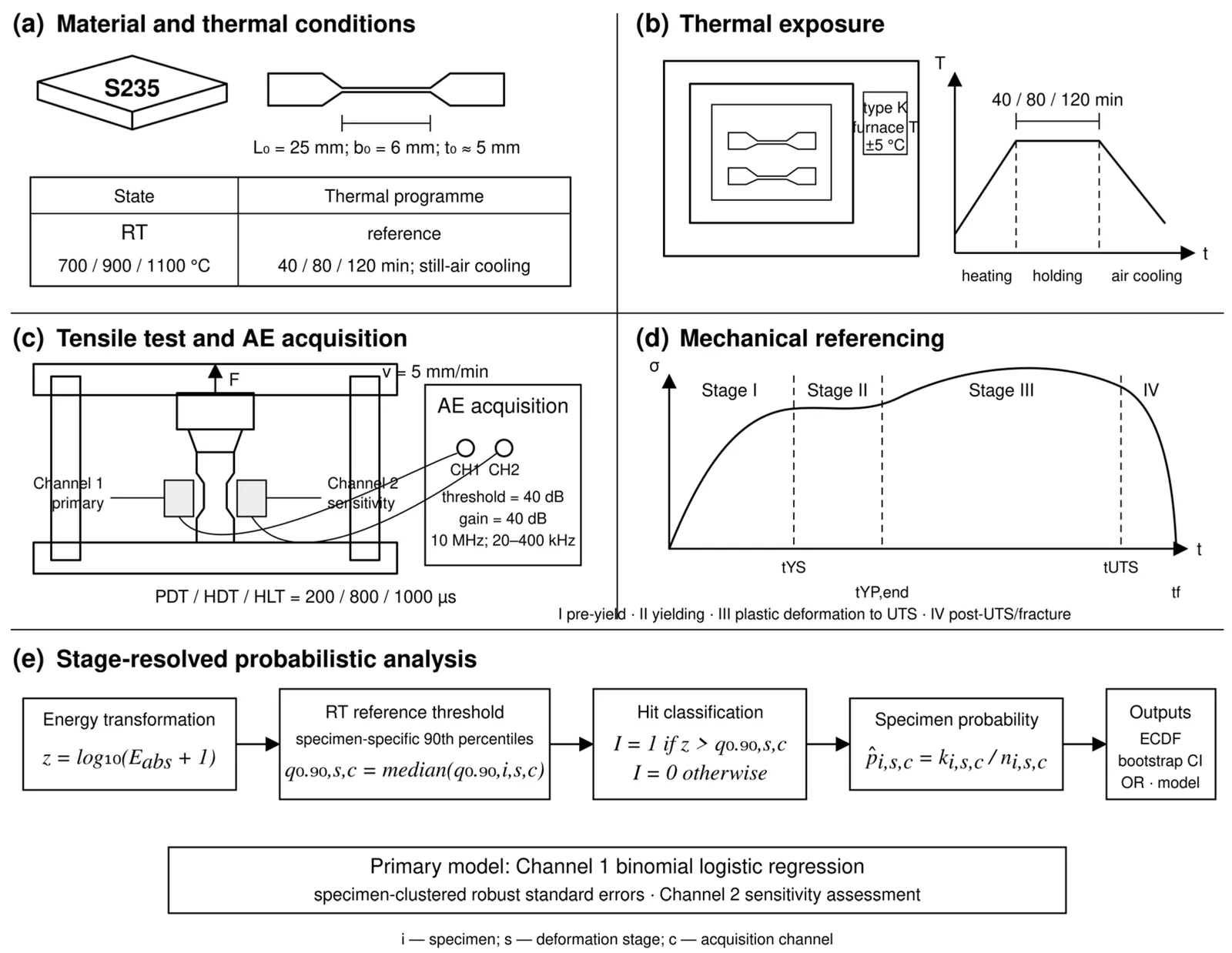
Experimental and probabilistic analysis workflow used in this study: (**a**) S235 steel material, specimen geometry, and investigated thermal conditions; (**b**) thermal exposure and subsequent still-air cooling; (**c**) tensile testing and two-channel acoustic-emission acquisition; (**d**) mechanical referencing of the AE record to four deformation stages; and (**e**) transformation of absolute AE energy, definition of stage- and channel-specific RT reference thresholds, classification of high-energy hits, calculation of specimen-level exceedance probabilities, and regression-based estimation of adjusted odds ratios. AE—acoustic emission; RT—room temperature; UTS—ultimate tensile strength; PDT—peak definition time; HDT—hit definition time; HLT—hit lockout time.

The experimental programme comprised a room-temperature reference condition and nine thermal-exposure conditions obtained by combining temperatures of 700, 900, and 1100 °C with holding times of 40, 80, and 120 min. Thermal exposure was performed in a chamber furnace with a temperature-control accuracy of approximately ±5 °C. The specimens were placed in the furnace at ambient temperature and heated together with the furnace. Holding time was measured from the moment at which the furnace reached the prescribed nominal temperature. After completion of the exposure period, the specimens were removed and cooled to ambient temperature in still laboratory air. The overall experimental and probabilistic analysis workflow is presented in Figure 1.

The furnace temperature was monitored using a type-K thermocouple positioned in the central region of the chamber. No thermocouples were attached directly to the specimens; therefore, the reported temperatures represent nominal furnace conditions rather than complete specimen-temperature histories. After cooling, the oxide scale was mechanically removed, and the specimen dimensions were measured again.

The full mechanical-testing programme comprised 30 completed tensile tests, nominally three per condition. Stage-resolved AE records were archived and suitable for hit-level processing for 20 specimens. The remaining 10 tests were excluded because no archived AE record was available; the exclusion was therefore based on data availability established before the present probabilistic analysis and not on the observed AE energy values or mechanical outcomes. This does not prove that missingness was random with respect to thermal condition, because record availability was unbalanced across the exposure matrix. The numbers available for the individual stages are summarized in Table 1, and Supplementary Table S1 accounts for all 30 tests, including AE-record status, usable channels and stages, and reasons for non-inclusion or incompleteness.

### 2.2. Uniaxial Tensile Testing

Uniaxial tensile tests were performed at ambient temperature using a Zwick/Roell universal testing machine (ZwickRoell GmbH & Co. KG, Ulm, Germany) equipped with a class 1 force-measurement system, in accordance with PN-EN ISO 6892-1:2020-05 [29]. The specimens were loaded continuously to fracture at a constant crosshead displacement rate of 5 mm/min. This value represents the machine-control parameter and not the strain rate measured directly within the gauge section.

Force and crosshead displacement were recorded throughout each test. Strain in the reduced section was measured using a mechanical extensometer with an initial gauge length of 25 mm and a displacement resolution of approximately 1–2 µm. Testing was performed at a laboratory temperature of 21 ± 1 °C and a relative humidity of approximately 40–50%.

Engineering stress was calculated as:

(1) $$\sigma =\frac{F}{A_{0}},$$

where F is the instantaneous tensile force and $A_{0}$ is the initial cross-sectional area. Engineering strain was calculated as:

(2) $$\varepsilon =\frac{\Delta L}{L_{0}},$$

where ΔL is the extensometer displacement and $L_{0}=25\;mm$ is the initial gauge length.

### 2.3. Acoustic Emission Acquisition

AE was recorded continuously using an Express-8 eight-channel acoustic-emission acquisition system controlled by AEwin software (MISTRAS Group, Inc., Princeton Junction, NJ, USA). Two VS75-SIC-40dB sensors (Vallen Systeme GmbH, Wolfratshausen, Germany) with integrated 40 dB preamplifiers were used. The sensors were mechanically secured, and technical silicone was used as the coupling medium.

Before each tensile test, sensor operation, coupling, and signal transmission were checked using the Hsu–Nielsen pencil-lead break procedure. Breaks were produced at reproducible locations relative to the sensors. This procedure was used as an operational verification and not as an absolute calibration of the recorded AE energy.

The principal acquisition settings were:

- detection threshold: 40 dB;
- integrated preamplifier gain: 40 dB;
- sampling frequency: 10 MHz;
- band-pass filter: 20–400 kHz;
- peak definition time: 200 µs;
- hit definition time: 800 µs;
- hit lockout time: 1000 µs.

The same acquisition configuration was applied to all specimens. The 40 dB threshold was selected above the background-noise level established during preliminary monitoring and retained unchanged throughout the programme. Consequently, only signals crossing this threshold were registered as AE hits.

Channel 1 was selected as the primary analysis path before fitting the probabilistic model because it contained the larger number of valid hits and provided the most complete common basis for stage-resolved comparisons (307,132 valid hits versus 128,040 for Channel 2). This selection was based on record completeness and not on the magnitude or statistical significance of the fitted effects. Channel 2 was analysed separately as a measurement-path sensitivity assessment, and its results are provided in the Supplementary Materials.

Absolute energy was obtained directly from the AE acquisition software and is reported in attojoules. Its recorded value depends on the complete measurement path, including the source–sensor distance, wave attenuation, specimen geometry, coupling, sensor response, amplification, and acquisition settings. Absolute energy was therefore treated as a configuration-dependent comparative descriptor rather than as an intrinsic material property.

### 2.4. Synchronization and Definition of Deformation Stages

Mechanical and AE datasets were exported and arranged on a common time axis. A constant offset was determined by aligning the onset of AE acquisition with the beginning of the increase in the mechanical force–time record. The synchronized records were matched with a temporal resolution of approximately 1 s. No local time stretching, nonlinear transformation, or stage-specific correction was applied.

Four characteristic times were identified separately for every specimen:

- $t_{YS}$: time corresponding to the lower yield point;
- $t_{YP,end}$: end of the yield plateau;
- $t_{UTS}$: time corresponding to the maximum engineering stress;
- $t_{f}$: final fracture time.

The end of the yield plateau was identified individually by inspection of the stress–time curve as the onset of a sustained stress increase associated with strain hardening. The same identification principle was applied to every specimen.

The AE record was divided into four mechanically defined stages:

$$\begin{array}{cc} Stage\;I: & \;t<t_{YS},\\ Stage\;II: & \;t_{YS}\leq t<t_{YP,end},\\ Stage\;III: & \;t_{YP,end}\leq t<t_{UTS},\\ Stage\;IV: & \;t_{UTS}\leq t\leq t_{f}. \end{array}$$

- t—synchronized test time, s.
- $t_{YS}$—time corresponding to the lower yield point, s.
- $t_{YP,end}$—time corresponding to the end of the yield plateau, s.
- $t_{UTS}$—time corresponding to the maximum engineering stress, s.
- $t_{f}$—time of final fracture, s.

Stage I represented the pre-yield period, Stage II the yield-plateau region, Stage III plastic deformation and strain hardening up to UTS, and Stage IV post-UTS localization, necking, and final fracture. This mechanical referencing limited comparisons between AE populations generated during fundamentally different portions of the tensile response.

### 2.5. Definition of a High-Energy AE Event

Because the absolute-energy distributions were strongly right-skewed, the recorded values were transformed as:

(3) $$E_{log}={log}_{10}\;\left(E_{abs}+1\right),$$

where $E_{abs}$ is the absolute energy reported by the acquisition software. Adding one allowed zero values to be retained.

A separate room-temperature reference threshold was established for every deformation stage and measurement channel. The 90th percentile was selected a priori as an upper-tail criterion that retained enough events for stable specimen-level binomial counts while distinguishing relatively energetic hits from the main detected population. First, the 90th percentile of the transformed absolute energy was calculated separately for each specimen, stage, and channel. This procedure gave equal weight to each RT specimen. The stage- and channel-specific threshold was then defined as the median of the specimen-level 90th percentiles:

(4) $$T_{s,c}={median}_{i}\;\left[Q_{0.90}\;\left(E_{log,i,s,c}\right)\right],$$

where i denotes the specimen, s the deformation stage, c the acquisition channel, and $Q_{0.90}$ the 90th percentile.

A hit was classified as high-energy when

(5) $$I_{i,s,c,j}=1\;\left(E_{log,i,s,c,j}>T_{s,c}\right),$$

where j indexes individual AE hits. The specimen-level high-energy exceedance probability was calculated as:

(6) $$P_{high,i,s,c}=\frac{H_{high,i,s,c}}{N_{i,s,c}}=\frac{1}{N_{i,s,c}}\sum_{j=1}^{N_{i,s,c}}I_{i,s,c,j},$$

where $H_{high,i,s,c}$ is the number of hits exceeding $T_{s,c}$ and $N_{i,s,c}$ is the total number of valid hits.

The resulting Channel 1 reference thresholds for Stages I–IV were 3.829, 3.150, 2.892, and 3.572, respectively, on the ${log}_{10}\left(E_{abs}+1\right)$ scale. The corresponding Channel 2 thresholds were 3.990, 2.822, 2.764, and 3.415.

### 2.6. Distributional and Descriptive Analyses

Empirical cumulative distribution functions were used to compare the distributions of $E_{log}$ between the reference material and the 40 min exposure conditions. The principal graphical comparison included RT, 700 °C/40 min, 900 °C/40 min, and 1100 °C/40 min because these groups provided the most balanced representation among the analysed conditions.

To prevent specimens or stages containing very large numbers of hits from dominating the ECDF visualizations, an equal-weight systematic sample of actual recorded hits was used. A maximum of 120 hits was retained for each specimen, channel, and stage. When more than 120 hits were available, observations were selected at approximately uniform positions from the chronologically ordered record; when 120 hits or fewer were available, all valid hits were retained. This subsampling was used exclusively for visualization. All available valid hits were used to calculate specimen-level quantiles, exceedance counts, and $P_{high}$.

For each thermal condition, stage, and channel, specimen-level probabilities were summarized using the median and observed minimum–maximum range. These ranges were used as the error bars in the specimen-level probability plots. Nonparametric percentile bootstrap intervals were calculated by resampling specimens with replacement within each condition. For the descriptive odds-ratio analysis, the examined condition and the corresponding RT group were resampled independently, and the odds ratio was recalculated from the resulting specimen-level medians. The bootstrap procedure used 10,000 resamples and a fixed random seed of 2026 to ensure reproducibility. Bootstrap intervals were reported only when at least three specimens were available in both the examined condition and the corresponding RT stage. Groups with fewer specimens were retained as exploratory observations.

Condition-specific odds ratios relative to RT were calculated from the median specimen-level probabilities:

(7) $$OR=\frac{P_{condition}/\left(1-P_{condition}\right)}{P_{RT}/\left(1-P_{RT}\right)},$$

Bootstrap confidence intervals for these descriptive odds ratios were reported only when sufficient specimen-level replication was available in both the examined condition and the corresponding RT stage. Missing confidence intervals were not replaced by zero.

### 2.7. Statistical Modelling and Cluster-Robust Inference

A binomial logistic model was fitted for Channel 1 to evaluate the association between thermal condition, deformation stage, exposure duration, and the occurrence of high-energy AE hits [28]. For each specimen–stage subset, the response was specified using the number of high-energy hits and the remaining number of valid hits. The model had the form

(8) $$logit\left(p\right)=\beta_{0}+\beta_{T}+\beta_{S}+\beta_{D}D,$$

where p is the probability of a high-energy hit, $\beta_{T}$ represents the categorical effect of exposure temperature, $\beta_{S}$ represents the categorical effect of deformation stage, and D is the exposure duration expressed per 80 min. RT and Stage I were used as the reference categories. No temperature-by-stage interaction was introduced because of the limited and unequal specimen representation across conditions.

Because multiple hit counts and deformation stages originated from the same specimen, conventional model-based standard errors would not adequately reflect within-specimen dependence [25,26,27]. Robust standard errors were therefore clustered by specimen. Statistical analyses were performed in Python 3.12 using NumPy 2.3.5 and SciPy 1.17.0. The cluster-robust sandwich covariance estimator was calculated using the specimen identifier as the clustering variable and included the finite-sample correction [*G*/(*G −* 1)] × [(*N −* 1)/(*N − K*)], where *G* is the number of specimen clusters, *N* is the number of specimen–stage observations, and *K* is the number of estimated model parameters. The fitted model contained 20 specimen clusters. Adjusted odds ratios were calculated as $exp\left(\beta \right)$, with 95% confidence intervals obtained from the specimen-clustered covariance matrix. Model-predicted probabilities and their 95% confidence intervals were subsequently calculated for every thermal condition and deformation stage.

The analysis was exploratory. Emphasis was placed on effect estimates, confidence intervals, agreement between descriptive and adjusted results, and the better-represented conditions rather than on isolated p-values. Channel 2 was not combined with Channel 1 or entered as an additional group in the principal model. It was evaluated separately to determine whether the broad stage-dependent patterns were reproduced through the second measurement path.

### 2.8. Sensitivity Analyses

Threshold sensitivity was evaluated by repeating the complete classification and Channel 1 regression procedure with RT reference thresholds defined at the 85th and 95th percentiles. For each percentile, specimen-specific RT quantiles, stage- and channel-specific median thresholds, exceedance counts, and the cluster-robust logistic model were recalculated.

Model stability was assessed using a leave-one-specimen-out analysis. Each of the 20 specimens was omitted in turn, after which the RT thresholds, hit classifications, and Channel 1 model were recalculated from the remaining data. Stability was evaluated from the direction and range of the adjusted odds ratios and from the number of refits in which the 95% confidence interval excluded unity. These analyses were intended to characterize sensitivity to the threshold definition and individual specimen clusters rather than to compensate for limited replication.

## 3. Results

### 3.1. Stage-Resolved Absolute-Energy Distributions

**Figure 2:**
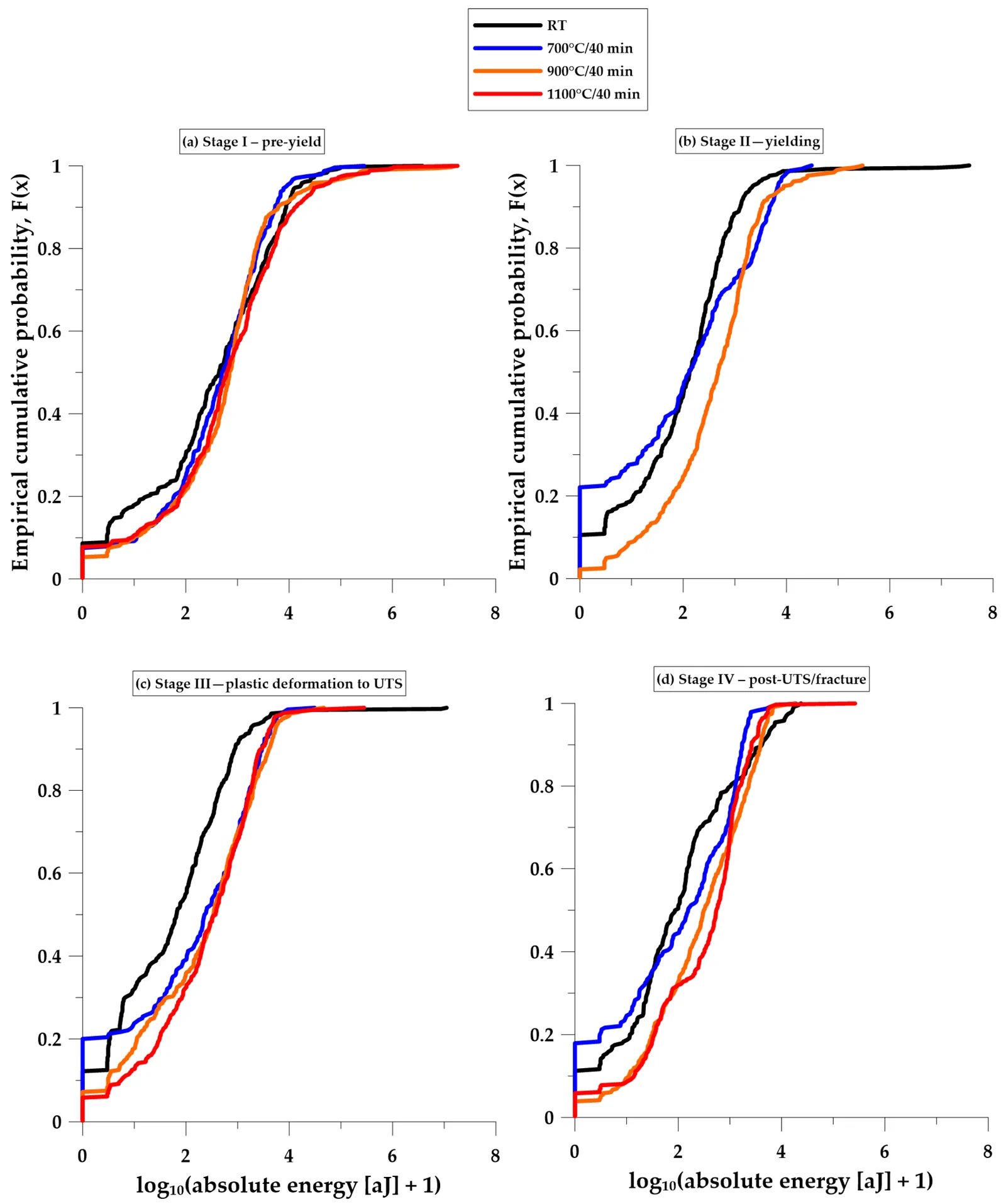
Empirical cumulative distribution functions of ${log}_{10}\left(E_{abs}+1\right)$ for acoustic-emission hits recorded by Channel 1 under the RT, 700 °C/40 min, 900 °C/40 min, and 1100 °C/40 min conditions: (**a**) Stage I—pre-yield; (**b**) Stage II—yielding; (**c**) Stage III—plastic deformation to UTS; and (**d**) Stage IV—post-UTS localization and fracture. The curves were constructed from equal-weight systematic samples of actual recorded hits. Subsampling was used exclusively for visualization.

Figure 2 presents the empirical cumulative distribution functions of ${log}_{10}\left(E_{abs}+1\right)$ recorded by Channel 1 for the RT, 700 °C/40 min, 900 °C/40 min, and 1100 °C/40 min conditions. The distributions differed between deformation stages and thermal conditions. The separation was comparatively limited during Stage I but became more pronounced during yielding and plastic deformation to UTS.

During Stage I, the median values of the equal-weight visualization samples ranged from 2.646 for RT to 2.848 for 900 °C/40 min. Differences were more apparent in the upper portions of the distributions. The corresponding 90th percentiles were 3.943 for RT, 3.729 for 700 °C/40 min, 3.789 for 900 °C/40 min, and 4.121 for 1100 °C/40 min.

During Stage II, the distributions for the thermally exposed specimens shifted toward higher values relative to RT, particularly above the median. The visualization-sample 90th percentile increased from 3.110 for RT to 3.675, 3.527, and 3.450 for the 700, 900, and 1100 °C/40 min conditions, respectively.

The clearest separation was observed during Stage III. The median ${log}_{10}\left(E_{abs}+1\right)$ value was 1.819 for RT and increased to 2.373, 2.535, and 2.574 after exposure to 700, 900, and 1100 °C for 40 min, respectively. The corresponding 90th percentiles were 2.948, 3.471, 3.606, and 3.454. Thus, all three thermally exposed conditions contained a larger proportion of observations in the upper part of the Stage III energy distribution than the RT condition.

During Stage IV, the distributions overlapped more strongly and did not follow a uniform temperature-dependent sequence. Their medians were 1.967 for RT, 2.193 for 700 °C/40 min, 2.502 for 900 °C/40 min, and 2.739 for 1100 °C/40 min, whereas the respective 90th percentiles were 3.623, 3.236, 3.591, and 3.417. The ECDF results therefore indicated that differences in central values and differences in the upper tail did not necessarily follow the same ordering.

### 3.2. Specimen-Level Probability of High-Energy Events

**Figure 3:**
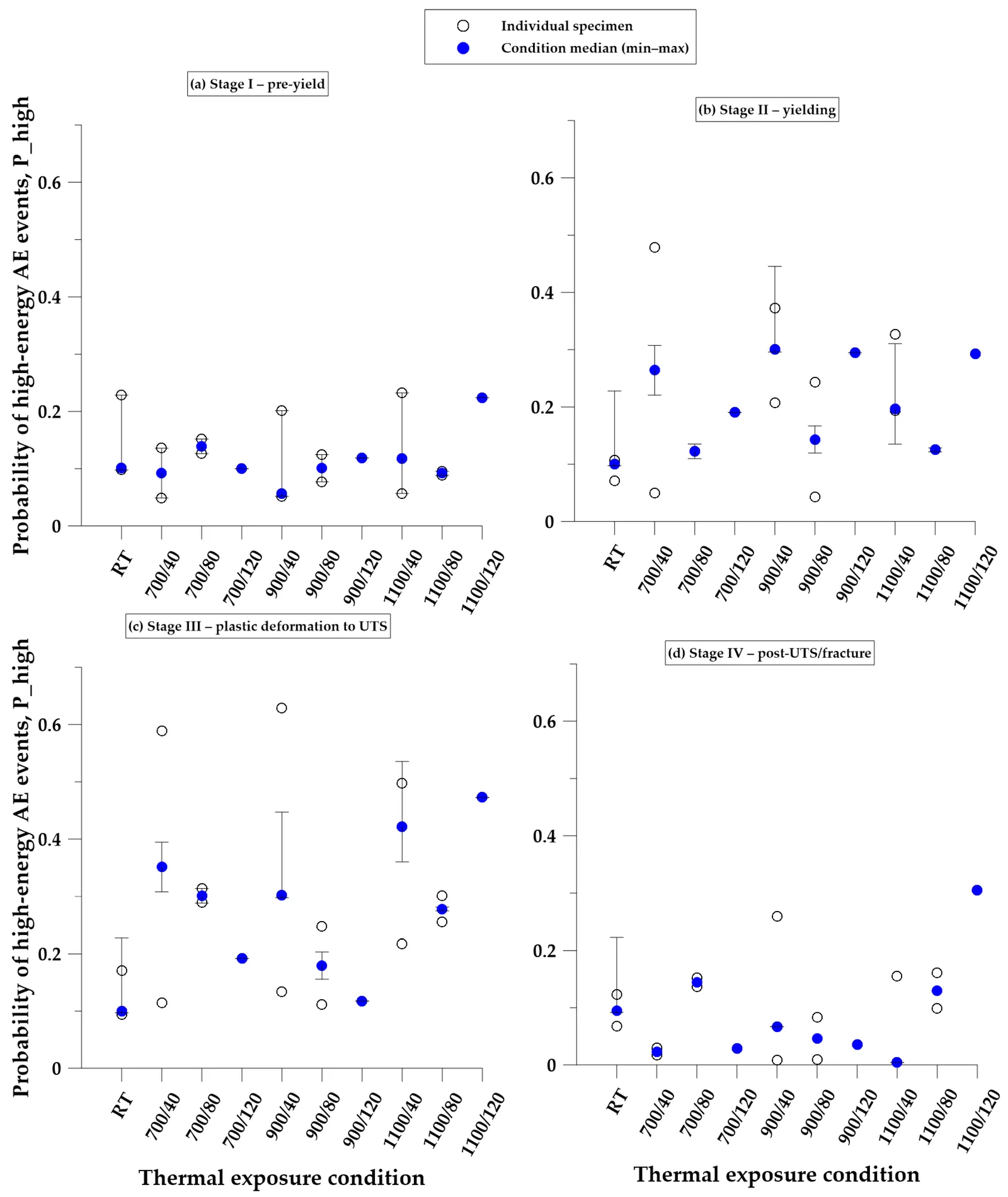
Specimen-level probabilities of high-energy acoustic-emission events recorded by Channel 1 for the investigated thermal-exposure conditions: (**a**) Stage I—pre-yield; (**b**) Stage II—yielding; (**c**) Stage III—plastic deformation to UTS; and (**d**) Stage IV—post-UTS localization and fracture. Open symbols represent individual specimens, filled symbols represent condition medians, and vertical bars show the observed minimum–maximum range. Single-specimen conditions are presented without ranges and should be interpreted as exploratory observations.

Figure 3 presents the specimen-level probability $P_{high}$ for Channel 1. By definition, the median RT probabilities remained close to 0.10 in Stages I–III, because the thresholds were derived from the specimen-specific RT 90th percentiles. The RT median in Stage IV was 0.095.

In Stage I, most median probabilities remained relatively close to the RT value. The medians for the 40 min conditions were 0.092 at 700 °C, 0.056 at 900 °C, and 0.118 at 1100 °C. The highest Stage I median was observed for the single available 1100 °C/120 min specimen ($P_{high}=0.224$); this value was retained as an exploratory observation.

Larger differences were observed during Stage II. The median probability increased from 0.100 for RT to 0.264, 0.300, and 0.196 after 40 min at 700, 900, and 1100 °C, respectively. Considerable between-specimen variation was present for some conditions. For example, the two Stage II values for 700 °C/40 min ranged from 0.050 to 0.478, whereas the three values for 900 °C/40 min ranged from 0.207 to 0.373.

Stage III produced the most consistent increases relative to RT. The RT median was 0.100, compared with 0.351 for 700 °C/40 min, 0.302 for 900 °C/40 min, and 0.422 for 1100 °C/40 min. Elevated Stage III probabilities were also observed for several longer-duration conditions, including medians of 0.301 for 700 °C/80 min, 0.278 for 1100 °C/80 min, and 0.473 for the single available 1100 °C/120 min specimen.

The Stage IV response differed from that observed during Stage III. The medians for the 700, 900, and 1100 °C/40 min conditions were 0.023, 0.067, and 0.004, respectively, compared with 0.095 for RT. Higher Stage IV values were observed for some longer-duration conditions, including 0.145 for 700 °C/80 min, 0.130 for 1100 °C/80 min, and 0.305 for the single 1100 °C/120 min specimen. Consequently, no monotonic relationship between exposure duration and $P_{high}$ was evident from the condition-level medians.

### 3.3. Condition-Specific Odds Ratios Relative to RT

**Figure 4:**
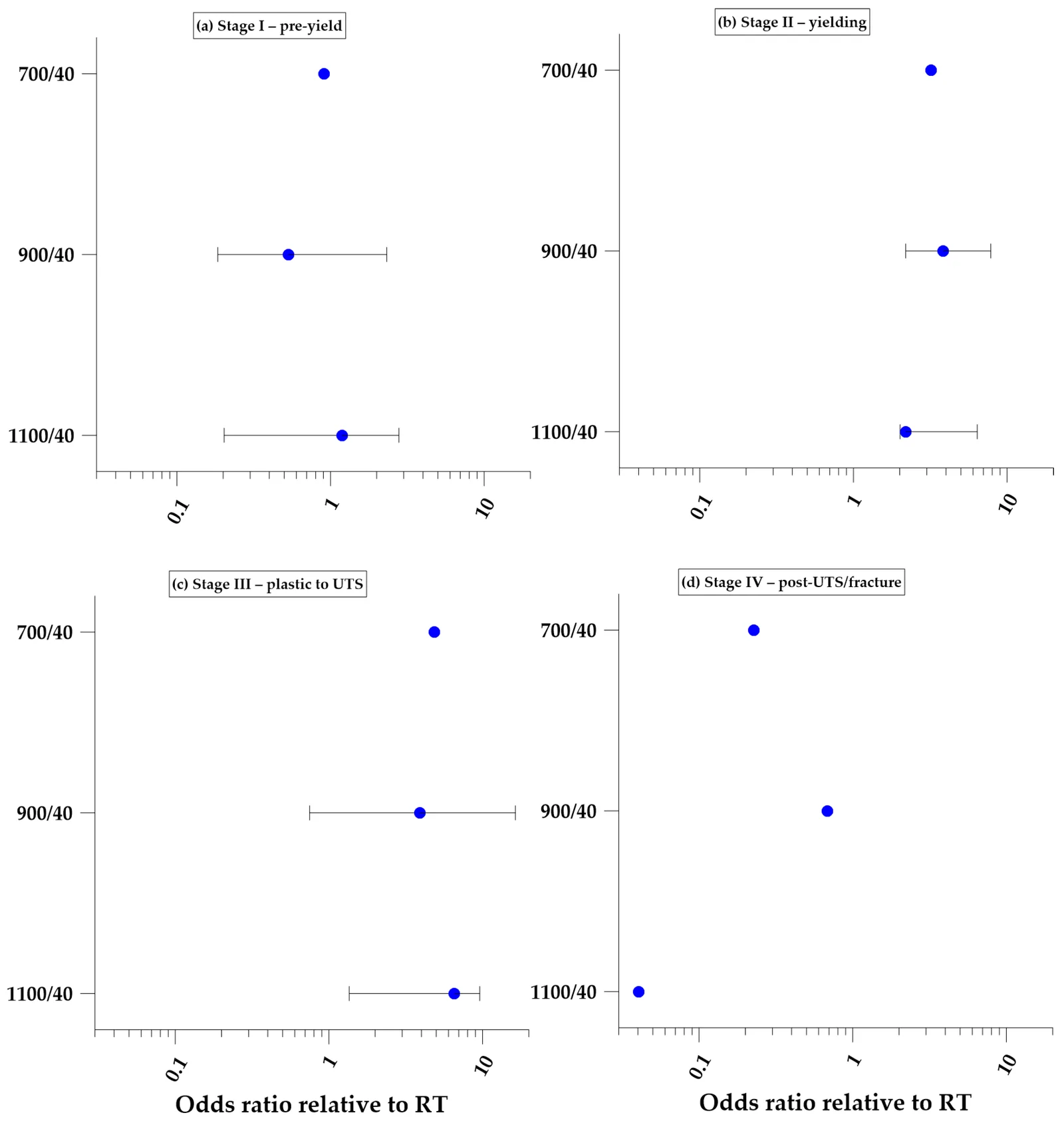
Odds ratios for high-energy acoustic-emission events recorded by Channel 1 after exposure for 40 min at 700, 900, and 1100 °C, relative to the room-temperature condition: (**a**) Stage I—pre-yield; (**b**) Stage II—yielding; (**c**) Stage III—plastic deformation to UTS; and (**d**) Stage IV—post-UTS localization and fracture. Points represent odds ratios calculated from condition medians; horizontal bars represent bootstrap 95% intervals where sufficient specimen-level replication was available. Intervals were not estimated for groups containing fewer than three specimens.

Figure 4 shows the descriptive odds ratios for the 40 min exposure conditions relative to RT. During Stage I, the odds ratios were 0.91 for 700 °C, 0.53 for 900 °C, and 1.19 for 1100 °C. The bootstrap intervals available for the 900 and 1100 °C conditions included unity, indicating substantial uncertainty in these comparisons.

During Stage II, the odds ratios increased to 3.21, 3.85, and 2.19 for the 700, 900, and 1100 °C/40 min conditions, respectively. The bootstrap 95% intervals were 2.18–7.83 for 900 °C/40 min and 2.01–6.39 for 1100 °C/40 min. No interval was calculated for 700 °C/40 min because only two specimens were available.

The largest descriptive odds ratios occurred during Stage III. Relative to RT, the odds ratios were 4.87 for 700 °C/40 min, 3.90 for 900 °C/40 min, and 6.56 for 1100 °C/40 min. The bootstrap interval was 0.75–16.37 for 900 °C/40 min and 1.35–9.59 for 1100 °C/40 min. The broad interval for 900 °C/40 min reflected substantial between-specimen variation.

During Stage IV, the corresponding odds ratios were below unity: 0.23 for 700 °C/40 min, 0.68 for 900 °C/40 min, and 0.04 for 1100 °C/40 min. Formal bootstrap intervals were unavailable because the Stage IV RT reference contained only two complete specimen records. The Stage IV comparisons should therefore be interpreted as descriptive.

### 3.4. Cluster-Robust Logistic Model Results

The fitted logistic model quantified the average associations of temperature, deformation stage, and exposure duration with the occurrence of high-energy hits in Channel 1 while accounting for clustering by specimen. The complete model results are presented in Table 2.

Relative to RT, exposure to 700 °C was associated with an adjusted odds ratio of 5.36 (95% CI: 1.64–17.59; p=0.0056). The adjusted odds ratio for 900 °C was 3.60 (95% CI: 0.93–13.93; p=0.0639), indicating a positive estimate accompanied by substantial uncertainty. Exposure to 1100 °C produced an adjusted odds ratio of 6.71 (95% CI: 3.03–14.88; p<0.001).

With Stage I used as the reference, the adjusted odds ratio was 2.61 for Stage II (95% CI: 1.64–4.15; p<0.001) and 3.54 for Stage III (95% CI: 2.15–5.84; p<0.001). Stage IV produced an odds ratio of 0.72 (95% CI: 0.35–1.48; p=0.366), providing no clear evidence of an overall difference from Stage I after adjustment for the other model terms.

The estimated odds ratio for an 80 min increase in exposure duration was 0.57 (95% CI: 0.18–1.79; p=0.339). Thus, the model did not identify a clear monotonic duration effect independent of temperature and deformation stage.

### 3.5. Threshold and Leave-One-Specimen-Out Sensitivity

Changing the RT reference threshold did not reverse the principal temperature or Stage II–III associations (Supplementary Table S3). At the 85th-percentile threshold, the adjusted odds ratios were 4.47 (95% CI: 1.45–13.77) for 700 °C, 2.89 (0.80–10.50) for 900 °C, and 5.57 (2.57–12.09) for 1100 °C. At the 95th-percentile threshold, the corresponding values were 6.48 (2.27–18.45), 4.07 (1.06–15.57), and 6.73 (2.89–15.70). Stage II and Stage III remained above Stage I at both alternative thresholds. The Stage IV estimate was more threshold-sensitive, ranging from 0.99 at the 85th percentile to 0.27 at the 95th percentile.

In the 20 complete leave-one-specimen-out refits, the Stage II, Stage III, and 1100 °C odds ratios retained positive directions and their 95% confidence intervals excluded unity in every refit (Supplementary Table S4). Their odds-ratio ranges were 2.25–3.86, 3.08–4.62, and 5.28–9.31, respectively. The 700 °C coefficient remained positive (OR range 3.03–12.58), but its confidence interval included unity in one refit. The 900 °C coefficient also remained positive (OR range 1.76–7.67), although its interval excluded unity in only 6 of 20 refits. Stage IV changed direction across refits. These findings support the stability of the broad Stage II–III and 1100 °C associations but reinforce the exploratory interpretation of the 700 °C, 900 °C, and Stage IV estimates.

### 3.6. Model-Predicted Probabilities

**Figure 5:**
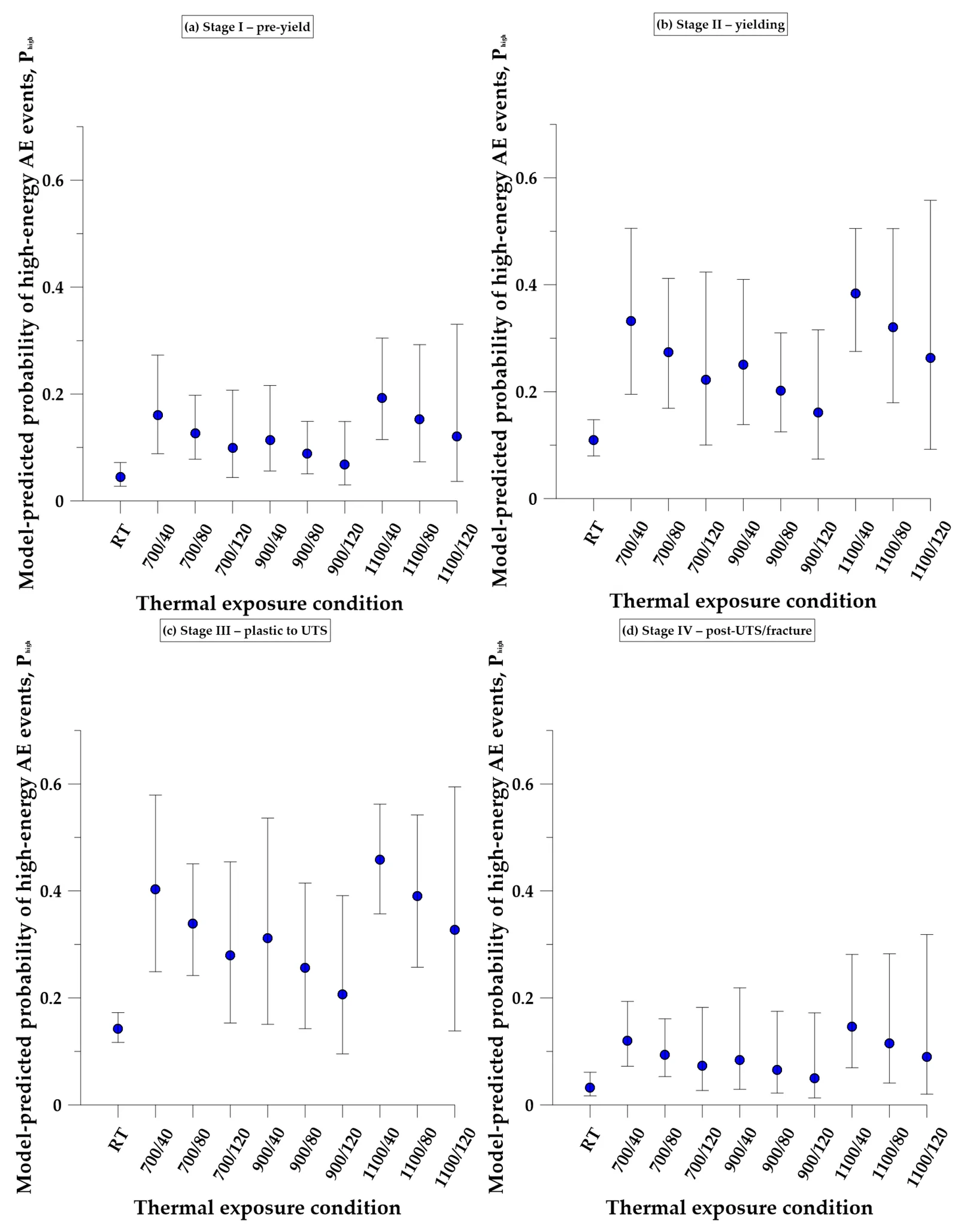
Model-predicted probabilities of high-energy acoustic-emission events recorded by Channel 1 for the investigated thermal-exposure conditions. Points represent predicted probabilities, and vertical bars indicate 95% confidence intervals. Panels correspond to: (**a**) Stage I—pre-yield; (**b**) Stage II—yielding; (**c**) Stage III—plastic deformation to UTS; and (**d**) Stage IV—post-UTS localization and fracture.

Figure 5 presents the probabilities predicted by the fitted model. For RT, the predicted probability increased from 0.045 in Stage I to 0.109 in Stage II and 0.142 in Stage III before decreasing to 0.033 in Stage IV.

For the 40 min exposure conditions, the highest predicted probabilities occurred consistently during Stage III. They were 0.403 for 700 °C, 0.312 for 900 °C, and 0.458 for 1100 °C. The corresponding Stage II probabilities were 0.332, 0.250, and 0.384. Predicted probabilities were lower in Stage I and Stage IV.

Within each temperature category, the additive model predicted progressively lower probabilities as exposure duration increased. However, the associated confidence intervals widened for the longer-duration conditions because these groups contained fewer specimens. For example, the predicted Stage III probability after exposure to 1100 °C decreased from 0.458 at 40 min to 0.390 at 80 min and 0.327 at 120 min, while the 95% confidence interval widened from 0.357–0.562 to 0.138–0.595 for the 120 min condition.

Because the model contained main effects without temperature-by-stage or temperature-by-duration interactions, its predictions represent smoothed average associations across the available records. They do not reproduce every condition-specific median. This distinction was most apparent in Stage IV, where several observed medians for the 40 min conditions were lower than the corresponding model predictions.

## 4. Discussion

### 4.1. Probabilistic Information Beyond Cumulative AE Activity

The principal finding is that the probability of a detected AE hit exceeding a stage-specific RT energy threshold varied with nominal furnace-exposure condition and deformation stage. This probability-based description complements cumulative counts and median hit parameters because it focuses on the upper part of the recorded energy distribution. The analysis establishes statistical associations in the measured AE response; it does not directly identify the physical source mechanism of individual hits.

The same experimental programme was previously examined using mechanically referenced cumulative AE counts [30]. That analysis addressed the timing and accumulation of threshold-crossing activity relative to characteristic points of the tensile response. The present study addresses a different question: whether thermal exposure changes the probability that an individual recorded hit exceeds a reference energy level within a given deformation stage. Consequently, cumulative counts are not reanalysed as the primary outcome, and the current results should be regarded as complementary rather than as a statistical reformulation of the earlier count-based study.

The distinction between cumulative activity and exceedance probability is important. A specimen may generate a large number of relatively low-energy hits and therefore exhibit high cumulative activity without a correspondingly high $P_{high}$. Conversely, a stage containing fewer hits may include a relatively large proportion of energetic observations. The probability framework retains this distinction and reduces the influence of the total number of hits when condition-level results are summarized at the specimen level.

### 4.2. Predominance of Stage III

The most consistent differences between the RT and thermally exposed conditions occurred during Stage III, covering plastic deformation from the end of the yield plateau to UTS. All three 40 min exposure conditions exhibited higher Stage III median probabilities than RT. Both the descriptive comparisons and the clustered model identified Stage III as the deformation period with the clearest increase in high-energy AE activity.

This pattern is consistent with the initial hypothesis that differences associated with nominal furnace exposure would be most visible during plastic deformation and strain hardening. Stage III involves sustained irreversible deformation before macroscopic localization becomes dominant, and several material or propagation-related processes may contribute to the recorded response [10,11,31]. However, the available scalar AE parameters do not permit individual signals to be assigned uniquely to dislocation motion, local yielding, microcrack development, oxide-related effects, or other potential sources [31,32]. The observed increase must therefore be interpreted as a statistical change in the recorded energetic AE population rather than direct evidence of a specific microstructural mechanism.

The results also demonstrate why mechanically defined stages are required. When all hits are analysed without reference to the tensile response, differences associated with Stage III can be diluted by the substantially different energy populations generated before yielding and after UTS. The stage-specific RT thresholds additionally account for the fact that the baseline energy distribution was not constant throughout the test.

### 4.3. Temperature and Exposure-Duration Effects

The clustered model indicated higher average odds of threshold exceedance following nominal furnace exposure, with the clearest estimates obtained for 700 and 1100 °C. The estimate for 900 °C was also positive but more uncertain. These results show associations with the nominal exposure-temperature categories; they neither establish a simple monotonic temperature dependence nor identify the metallurgical cause of the recorded differences.

Exposure duration produced no clear independent monotonic association. The estimated odds ratio per 80 min was 0.57, but its confidence interval was wide and included unity. Condition-specific medians also varied irregularly with duration. These findings are consistent with the expectation that the residual state of thermally exposed steel depends on the combined temperature–duration condition rather than on either variable considered separately [1,2,3,4]. Nevertheless, the unequal specimen representation at 80 and 120 min prevents strong conclusions concerning the functional form of the duration effect.

The lower model-predicted probabilities at longer exposure durations arise from the fitted negative duration coefficient. They should not be interpreted as proof that longer exposure systematically reduces energetic AE activity. The estimates for the longer-duration conditions were based on fewer specimens and were accompanied by wider confidence intervals. Moreover, the additive model did not include temperature-by-duration or temperature-by-stage interactions.

### 4.4. Difference Between Descriptive and Model-Based Results

Some differences were observed between the specimen-level medians and the probabilities predicted by the logistic model, particularly during Stage IV. This is not a computational inconsistency. The descriptive analysis assigned equal importance to each available specimen when calculating a condition median. By contrast, the binomial model used the numbers of high-energy and remaining hits, so specimen–stage subsets containing more hits contributed more information to the coefficient estimates. Cluster-robust standard errors accounted for dependence within specimens but did not make the point estimates identical to equally weighted specimen medians.

The model also estimated common temperature and stage effects across the complete dataset. It therefore smoothed local differences between individual temperature–duration–stage combinations. For example, the very low Stage IV median observed after exposure to 1100 °C for 40 min was not reproduced directly by the corresponding model prediction because the positive overall coefficient for 1100 °C was estimated across all four stages. An interaction model could describe such condition-specific differences more flexibly, but the available number of specimen clusters was insufficient to support a substantially more complex parameterization.

For this reason, the descriptive probabilities and adjusted model should be interpreted together. The model estimates broad average associations while accounting for clustered uncertainty, whereas the specimen-level plots reveal local heterogeneity that the additive model cannot represent.

### 4.5. Stage IV and Post-UTS Behaviour

Stage IV did not show the same consistent increase as Stage III. For the 40 min conditions, the descriptive median probabilities were below the RT value, with the lowest value observed for 1100 °C/40 min. In contrast, some 80 and 120 min conditions showed renewed increases. The clustered model consequently produced no clear overall difference between Stage IV and Stage I.

Changes in AE characteristics during the post-UTS period have previously been associated with the transition from distributed plastic deformation to localized necking and final fracture [11,12,13,15,33]. The accompanying modification of the propagation path and concentration of deformation within a progressively smaller region may additionally influence the waveforms detected by each sensor [17,18,19,21,22]. Accordingly, the Stage IV results may reflect both changes in active material processes and changes in the geometry and propagation path occurring during localization.

Stage IV also differed considerably in duration and number of recorded hits between specimens. In addition, not every specimen provided a complete post-UTS record. These factors increase uncertainty and make Stage IV particularly sensitive to individual-test behavior. The Stage IV results should therefore be treated as exploratory and should not be used alone to rank the thermal conditions.

### 4.6. Sensitivity to the Measurement Channel

Channel 2 was evaluated separately because the two sensors recorded the response through different propagation paths. The supplementary results reproduced the presence of stage- and condition-dependent variation, but they did not reproduce every Channel 1 ordering or absolute probability. The paired condition-level summaries for both channels are provided in Supplementary Table S2. This outcome is expected because AE energy depends on source location, propagation distance, attenuation, coupling, sensor response, and the evolving geometry of the specimen [17,18,19,20,21,22].

The lack of identical values between channels does not necessarily indicate unreliable acquisition. Instead, it confirms that absolute-energy exceedance is dependent on the measurement path. The channel-specific RT thresholds reduced, but could not eliminate, this dependence. Combining the channels would have obscured this distinction and could also have counted the same physical source activity more than once. The present analysis therefore treats Channel 1 as the primary measurement path and Channel 2 as a sensitivity assessment rather than as a technical replicate.

The supplementary Channel 2 results are consequently important for evaluating robustness. They indicate that the broad conclusion—namely that energetic AE activity varies across deformation stages and thermal conditions—was not restricted to a single sensor. At the same time, the differences between channels show that the numerical probabilities and thresholds should not be transferred directly to another sensor arrangement.

### 4.7. Implications for AE-Based Assessment

A probability of exceeding a reference energy level is potentially useful because it can be interpreted more directly than a cumulative energy sum dominated by a small number of extreme hits. It also provides a dimensionless specimen-level descriptor that can be compared between mechanically equivalent stages. In the present experiments, $P_{high}$ distinguished several thermally exposed conditions most clearly during yielding and plastic deformation to UTS.

The proposed thresholds are internal reference values derived from the investigated RT specimens. Their numerical values depend on the acquisition system, threshold, amplification, sensor characteristics, coupling, specimen geometry, and signal-propagation conditions. The method is therefore transferable primarily as an analysis framework: reference distributions should be established separately for a given measurement configuration.

The 40 dB detection threshold also truncates the observable AE population because only signals exceeding that amplitude are registered as hits [18,20]. A constant threshold improves consistency among tests but prevents conclusions about activity below the acquisition boundary. The calculated probabilities therefore refer to high-energy events within the population of detected hits, not within the unknown population of all physical emission sources.

At its present stage, the approach should be regarded as a material-level comparative method rather than a field-ready post-fire classification procedure. Application to structural components would require validation under variable sensor spacing, attenuation, structural geometry, restraint, loading history, and background noise.

### 4.8. Limitations and Further Work

The principal limitation is the unequal and generally small number of specimens available for the hit-level analysis. Although the complete mechanical programme comprised 30 specimens, the probabilistic dataset contained 20 specimen clusters, and several 80 and 120 min conditions were represented by only one or two specimens. Estimates for these groups were retained to document the available observations, but they cannot provide stable condition-specific inference. The conclusions should therefore focus primarily on the broad stage-related effects and on the better-represented RT, 900 °C/40 min, and 1100 °C/40 min groups.

A second limitation concerns the logistic model. Twenty specimen clusters permit estimation of a compact exploratory model, but not a full set of temperature-by-duration-by-stage interactions [25]. The additive structure was selected to avoid excessive parameterization. The complete leave-one-specimen-out analysis preserved the positive Stage II, Stage III, and 1100 °C associations in all refits, but the precision of the 700 and 900 °C estimates was more sensitive to individual specimens. These results improve transparency regarding model stability but do not replace balanced independent replication.

Third, the high-energy classification depends on an internally defined percentile threshold. Repeating the complete analysis at the 85th and 95th percentiles preserved the positive temperature coefficients and the higher Stage II–III odds, supporting the qualitative robustness of these findings. The Stage IV coefficient was less stable across thresholds and leave-one-specimen-out refits. The percentile criterion remains analytical rather than physically universal, and the resulting numerical probabilities should not be transferred to another measurement configuration without a new reference distribution.

Fourth, no direct quantitative correlation with chemical composition or thermally modified microstructure was available for the present dataset. The observed AE differences cannot therefore be assigned conclusively to grain growth, phase transformations, recovery, recrystallization, changes in dislocation structure, or residual stresses. Future studies should combine the probability-based AE analysis with quantitative metallography, hardness, and phase characterization.

Finally, the specimens were tested using one geometry, loading rate, acquisition system, sensor type, and coupling arrangement. Further validation should include additional S235 products and steel grades, balanced specimen numbers, repeated sensor layouts, structural-scale elements, and realistic heating and cooling histories. Such work would establish whether probability-based AE descriptors can support reliable classification of thermally modified steel beyond the controlled laboratory configuration used here.

## 5. Conclusions

This study evaluated the probability of recording high-energy AE events during mechanically defined stages of tensile deformation in thermally exposed S235 structural steel. The following conclusions were drawn:

1. For the investigated specimens, the exceedance probability varied across nominal furnace-exposure conditions and mechanically defined deformation stages. This is an association in the recorded AE response and not direct evidence of a particular microstructural mechanism.
2. The clearest and most stable stage-related result was the higher adjusted odds in Stage III relative to Stage I. Stage II was also consistently higher than Stage I across the threshold and leave-one-specimen-out analyses.
3. The adjusted analysis indicated higher overall odds after nominal exposure at 700 and 1100 °C. The 1100 °C association remained supported in every leave-one-specimen-out refit. The 700 °C direction was stable, but its confidence interval included unity in one refit; the positive 900 °C estimate was more uncertain. No clear monotonic exposure-duration association was identified.
4. Stage IV exhibited greater condition-specific variability and was sensitive to both the percentile threshold and individual specimens. It should therefore be interpreted as exploratory, particularly for sparsely represented conditions.
5. Channel 2 confirmed the general presence of stage- and condition-dependent variation but did not reproduce all Channel 1 rankings or absolute probabilities, demonstrating the influence of the measurement and propagation path.

The proposed exceedance-probability framework complements cumulative AE measures and central hit descriptors by quantifying changes in the upper part of the energy distribution. The numerical thresholds obtained here are specific to the investigated material and acquisition configuration. Balanced replication and validation using additional steel products and measurement arrangements are required before general diagnostic criteria can be established.

## Figures and Tables

**Table 1:** Thermal-exposure matrix and numbers of specimens included in the Channel 1 stage-resolved probabilistic analysis.

Condition	Temperature (°C)	Holding Time (min)	Specimens Mechanically Tested	Stage I	Stage II	Stage III	Stage IV
RT	RT	—	3	3	3	3	2
700 °C/40 min	700	40	3	2	2	2	2
700 °C/80 min	700	80	3	2	2	2	2
700 °C/120 min	700	120	3	1	1	1	1
900 °C/40 min	900	40	3	3	3	3	3
900 °C/80 min	900	80	3	2	2	2	2
900 °C/120 min	900	120	3	1	1	1	1
1100 °C/40 min	1100	40	3	3	3	3	3
1100 °C/80 min	1100	80	3	2	1	2	2
1100 °C/120 min	1100	120	3	1	1	1	1

**Table 2:** Cluster-robust binomial logistic model for high-energy AE events recorded by Channel 1.

Predictor	Coefficient, β	Cluster-Robust SE	Adjusted OR	95% CI for OR	p-Value
Intercept	−3.060	0.255	0.047	0.028–0.077	<0.001
Temperature: 700 °C	1.680	0.606	5.36	1.64–17.59	0.0056
Temperature: 900 °C	1.280	0.691	3.60	0.93–13.93	0.0639
Temperature: 1100 °C	1.903	0.406	6.71	3.03–14.88	<0.001
Stage II: yielding	0.960	0.236	2.61	1.64–4.15	<0.001
Stage III: plastic deformation to UTS	1.264	0.255	3.54	2.15–5.84	<0.001
Stage IV: post-UTS/fracture	−0.333	0.369	0.72	0.35–1.48	0.366
Exposure duration, per 80 min	−0.554	0.580	0.57	0.18–1.79	0.339

Reference categories: RT and Stage I. Confidence intervals and p-values were calculated using standard errors clustered by specimen.

## Data Availability

The original contributions presented in this study are included in the article and the Supplementary Materials. Further inquiries can be directed to the corresponding author.

## Supplementary Materials

The following supporting information can be downloaded at: https://www.mdpi.com/article/10.3390/ma19214602/s1, Table S1: Accounting of all 30 mechanically tested specimens and availability of stage-resolved AE records; Table S2: Paired condition-level summaries of stage-resolved high-energy-event exceedance probabilities for Channels 1 and 2; Table S3: Sensitivity of the Channel 1 cluster-robust logistic model to 85th-, 90th-, and 95th-percentile RT thresholds; Table S4: Leave-one-specimen-out stability summary for the complete 90th-percentile analysis pipeline; Dataset S1: Specimen-level data for the primary 90th-percentile analysis; Dataset S2: Specimen-level counts and exceedance probabilities for the threshold-sensitivity analysis; Dataset S3: Coefficient estimates from all leave-one-specimen-out refits.
